# CiliaKB: A comprehensive knowledge base for cilia-associated genes

**DOI:** 10.1371/journal.pbio.3003774

**Published:** 2026-04-22

**Authors:** Chun-Jie Liu, Chao Zhang, Wei Feng, Gaoxiang Huang, Xiaopeng Zou, Wenlu Wei, Donghui Zhang

**Affiliations:** 1 Zhanjiang Institute of Clinical Medicine, Central People’s Hospital of Zhanjiang, Guangdong Medical University Zhanjiang Central Hospital, Zhanjiang, PR China; 2 Center for Artificial Intelligence Biology, Hubei Bioinformatics & Molecular Imaging Key Laboratory, Key Laboratory of Molecular Biophysics of the Ministry of Education, College of Life Science and Technology, Huazhong University of Science and Technology, Wuhan, PR China

## Abstract

Cilia dysfunction is implicated in a range of disorders. This Community Page presents CiliaKB, a manually curated knowledge base that serves as a one-stop platform for researchers to rapidly access mechanistic data and mine for translational clues about cilia.

## Teaser

Cilia dysfunction is implicated in a range of disorders. Here, we present CiliaKB, a manually curated knowledge base that serves as a one-stop platform for researchers to rapidly access mechanistic data and mine for translational clues about cilia.

Cilia, microtubule-based organelles protruding from the surface of most mammalian cells, have long been recognized as important for a myriad of cellular processes. These structures are involved in sensory perception, signal transduction, the regulation of cell motility, and other vital functions. The processes of ciliogenesis and the maintenance of the structure and function of cilia are strictly programmed and precisely modulated by a series of proteins [1,2]. Dysfunction of cilia is now known to underlie a broad spectrum of disorders, collectively termed ciliopathies, which include diseases such as polycystic kidney disease, Bardet–Biedl syndrome, and primary ciliary dyskinesia. Beyond ciliopathies, ciliary dysfunction has also been implicated in the pathogenesis of various cancers [3], neurodegenerative diseases [4], and other systemic disorders such as idiopathic scoliosis [5], underscoring the far-reaching impact of these organelles on human health.

In recent years, advancements in genomic sequencing, proteomics, and imaging technologies have led to a surge in the identification of genes associated with cilia structure and function [6,7]. Surprisingly, current studies have revealed that the number and diversity of cilia-associated genes are far greater than previously estimated. This expansion in our understanding of the ciliary proteome has, however, brought a significant challenge: the fragmentation of information across numerous publications, databases, and research platforms. This fragmentation hinders the ability of researchers to integrate data from different sources, limiting our capacity to grasp the full complexity of ciliary biology, including the intricate networks of inter-organelle crosstalk, the physiological roles of ciliary genes in various tissues and developmental stages, and their contributions to pathogenesis.

Against this backdrop, there is an urgent need for a comprehensive, centralized resource that integrates systems biology approaches, inter-organelle regulatory networks, physiological functions, and disease associations of ciliary genes. Such a resource would not only facilitate basic research into cilia biology but also accelerate translational efforts aimed at developing novel diagnostic and therapeutic strategies for cilia-related diseases. We produced CiliaKB as a response to this critical need: a comprehensive and manually curated knowledge base specifically designed to focus on cilia-associated genes and their roles in organelle crosstalk, physiological processes, and diseases. By offering a unified and expanded platform that integrates diverse dimensions of ciliary biology, CiliaKB aims to bridge the existing gap in our understanding and provide researchers with a powerful tool to advance the field.

## What is CiliaKB?

**Fig 1:**
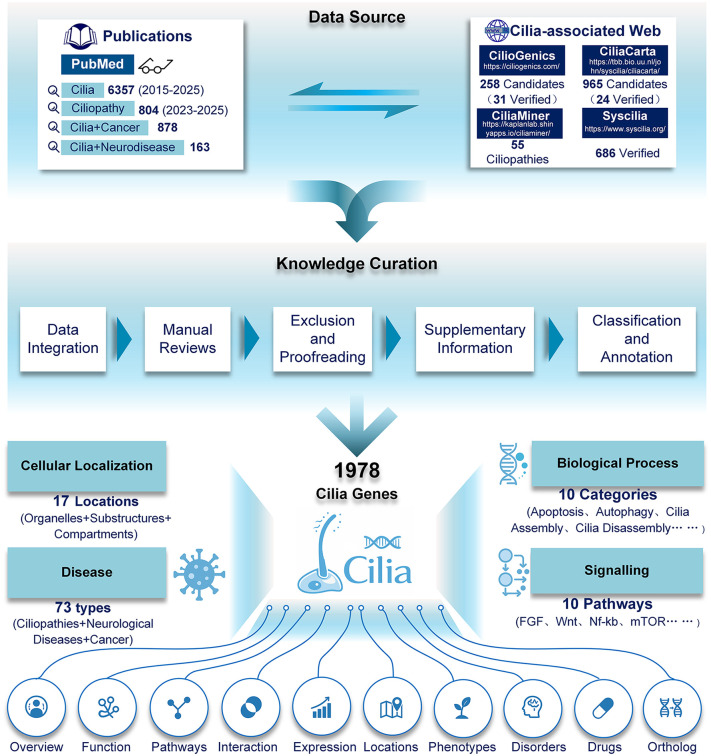
General overview of the CiliaKB construction and content. The database integrates 1,978 genes with multi-dimensional annotations based upon previous resources such as SYSCILIA gold standard version 2 (686 genes), CiliaCarta (965 candidates (24 newly validated genes)), and CilioGenics (258 candidates (31 newly validated genes)), and is supported by evidence from more than 6,000 publications, including specialized subsets focused on ciliopathies, cancers, and neurological diseases.

CiliaKB provides a manually curated and significantly expanded list of 1,978 cilia-associated genes., including both first-order cilia genes (those that principally localize to, and function within, the basal body and/or the ciliary compartment) and second-order cilia genes (not localized within cilia but have a role in cilium formation or function) that control ciliary structure and function, but not candidate cilia-associated genes. Each gene is annotated based on rigorous evidence extracted from over 6,000 publications and existing databases of cilia (an updated SYSCILIA gold standard (SCGSv2): 686 validated genes [8]; CiliaCarta: 965 candidates (24 newly validated genes) [9]; and CilioGenics: 258 candidates (31 newly validated genes) [10]). We searched PubMed and retrieved information on 6,357 articles using the keyword “cilia” (2015–2025), 804 articles using the keyword “ciliopathy” (2023–2025), 878 articles using the keyword “cilia and cancer”, and 163 articles using the keyword “cilia+neurodisease” (Fig 1). Our curated references for cilia genes mainly cover works published from 2015 onward. This starting year was chosen based on the release timelines of two key resources just prior to 2015: the SYSCILIA Gold Standard (first version in 2013, major update in 2021) and Cildb (published in 2014), which together captured most known cilia genes up to that time. However, our knowledge about cilia has since expanded significantly, with extensive data being generated after 2015. Indeed, many newly identified cilia-related genes, such as *ZNF703* (*nlz1*) (2015), *MICALL1* (2019), and *FFAR4* (2019), are still absent from the updated SYSCILIA Gold Standard (2021), and Cildb has not been updated since 2014.

This curation process ensures that the included genes are supported by robust experimental data, ranging from genetic studies to proteomic analyses. To enhance the utility of this gene list, each entry is accompanied by detailed functional domain information, which will help researchers understand the molecular mechanisms underlying the function of each ciliary protein. Recognizing the importance of user-friendly access, CiliaKB also features interactive interfaces that support advanced search capabilities, allowing researchers to filter genes based on various criteria such as localization or disease association. Furthermore, visualization tools help illustrate complex relationships between genes and their associated pathways.

## Setting cilia-associated genes in a wider context

**Fig 2:**
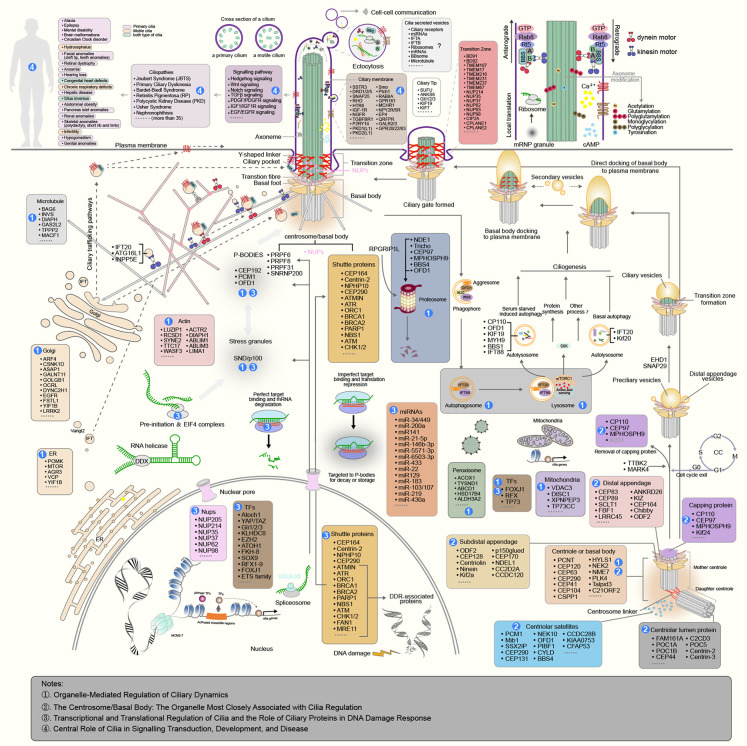
Cilia atlas in the cell. ① Organelle-Mediated Regulation of Ciliary Dynamics. Formation of primary cilia physically and functionally integrates with diverse intracellular organelles, not just the centrosome/basal body. Multiple organelles coordinate to regulate ciliogenesis and cilia function and structure through specialized mechanisms. For example, the centrosome-derived basal body serves as the microtubule-organizing center for axoneme nucleation. The endoplasmic reticulum establishes membrane contact sites at the ciliary base to control Ca²⁺-dependent signaling and lipid transfer. And mitochondria hold a crucial supportive role in cilia formation by providing the necessary energy in the form of ATP, regulating mitochondrial transcription, calcium signaling, and redox balance for the assembly and maintenance of cilia. ② The Centrosome/Basal Body. Ciliogenesis initiates with the docking of a preciliary vesicle (PCV) onto the mother centriole’s distal end, mediated by distal appendages. The emerging transition zone (TZ) then invaginates the ciliary vesicle as it expands through vesicle fusion. The basal body–vesicle complex subsequently docks to and fuses with the plasma membrane, enabling TZ maturation and axoneme elongation—a process driven by IFT (Intraflagellar Transport) and BBS (Bardet–Biedl Syndrome) complexes. Some cells bypass ciliary vesicle formation and directly dock the basal body to the membrane. ③ Transcriptional and Translational Regulation of Cilia and the Role of Ciliary Proteins in DNA Damage Response. **(a)** Transcriptional control of ciliogenesis. Key transcription factors (e.g., FOXJ1, RFX family) regulate the expression of ciliary genes. Their activity is modulated by cell cycle status, extracellular signals, and post-translational modifications. **(b)** Translational regulation in cilia assembly. RNA-binding proteins and miRNAs localize to the cytoplasm and the basal body, where they control the spatial translation of specific mRNAs required for ciliary construction and maintenance. **(c)** DNA damage response mediated by ciliary proteins. Several ciliary proteins (e.g., CEP164, CEP290) translocate to sites of DNA damage and participate in repair complex assembly. Ciliary dysfunction leads to impaired DNA damage response and genomic instability. ④ Central Role of Cilia in Signaling Transduction, Development, and Disease. The ciliary membrane is enriched with a variety of receptors, such as G protein-coupled receptors and receptor tyrosine kinases. These receptors are involved in sensing and transducing extracellular signals, regulating key pathways including Hedgehog, Wnt, and PDGF signaling. Disruption of these ciliary signaling pathways, due to genetic mutations or functional impairments, can lead to ciliopathies, tumors, neurodegenerative diseases, and other diseases.

CiliaKB features a systematic and manual classification of cilia-associated genes based on their subcellular localization and cellular process. This classification provides valuable insights into the spatial organization of the ciliary machinery and is aimed at helping researchers understand how different proteins contribute to cilia structure and function. Beyond ciliary proteins themselves, CiliaKB summarizes the ciliary relevance of nonciliary genes localized in other cellular compartments, including the nucleus, cytoplasm, cytoskeleton, mitochondria, Golgi apparatus [11], endoplasmic reticulum [11], and peroxisome (Fig 2). This broader perspective is crucial because cilia do not function in isolation, but are part of a complex network of inter-organelle communication. For example, genes localized in the nucleus may regulate the transcription of ciliary components, while mitochondrial genes may influence ciliary function through energy metabolism (Fig 2).

CiliaKB systematically integrates evidence demonstrating the multifaceted roles of cilia and ciliary proteins in regulating various cellular processes such as the DNA damage response [12], cellular senescence [4], cell division, and autophagy. Moreover, CiliaKB highlights the role of cilia and ciliary proteins as critical signaling hubs that coordinate more than a dozen key cellular signaling pathways, including FGF, Hippo, Wnt, PDGF, and mTOR [13]. By connecting these processes and pathways to cilia-related disease mechanisms, the knowledge base provides a systems-level view of how ciliary defects contribute to various diseases.

Notably, while cilia modulate diverse biological and cellular signaling pathways, their own structure and function are reciprocally controlled by multiple cellular pathways, indicating a sophisticated, bidirectional regulatory network. Through integrated multi-omics data and functional interaction networks, CiliaKB offers a comprehensive resource for exploring how cilia achieve cross-pathway integration and context-specific signal modulation. This integrated view could help researchers uncover novel regulatory mechanisms and identify potential therapeutic targets that might otherwise be overlooked.

## Using CiliaKB for translational research

To illustrate the diverse roles of cilia in pathogenesis, CiliaKB provides detailed gene annotations across a wide range of disorders, including ciliopathies (based on Ciliaminer [14]), cancers, neurodegenerative diseases, and other systemic disorders. For example, the knowledge base allows researchers to explore how ciliary gene expression varies across different tissues and developmental stages. This information could be particularly useful for understanding the tissue-specific manifestations of cilia-related diseases. Additionally, CiliaKB includes data on known mutations in ciliary genes, along with their associated phenotypes, enabling the establishment of phenotype–genotype links. Understanding these links will be critical for improving the diagnosis of cilia-related diseases. By providing access to this omics data through interactive interfaces, CiliaKB aims to empower researchers to identify potential biomarkers, develop predictive models of disease progression, and design personalized therapeutic strategies. We hope that this integration of omics data with functional and disease-related annotations will make CiliaKB a powerful tool for bridging the gap between basic research and clinical application.

## Conclusions

CiliaKB provides a comprehensive, manually curated knowledge base that integrates diverse aspects of ciliary gene function, regulation, and disease association. Its comprehensive gene collection, systematic classification based on subcellular localization, cell processes, detailed disease annotations, and interactive omics features make it a valuable resource for researchers and clinicians. By addressing the fragmentation of ciliary research data, we hope that CiliaKB can accelerate our understanding of cilia biology, allowing researchers to uncover novel regulatory mechanisms and facilitate the development of improved diagnostic and therapeutic approaches for cilia-related diseases. As research into cilia continues to progress, CiliaKB could hold a central role in synthesizing new findings and driving innovation in the field. Future developments of CiliaKB will include the integration of real-time updates based on newly published research, further enhancing its utility and ensuring its continued relevance for cilia research.

## Abbreviations

NSFC: National Natural Science Foundation of China
PCV: preciliary vesicle
TZ: transition zone.
